# Progress in Disease of Digestive Organs

**Published:** 1902-03-29

**Authors:** 


### Progress in Disease of Digestive Organs.

**Gastric Ulcer.**—The cause is said, by Van Ijzeren,[1] to be muscular spasm obliterating the vessels of the mucous membranes. This spasm is a reflex due to the passage of food through the duodenum. He succeeded in producing typical ulcers by subdiaphragmatic division of the vagus, and their occurrence can be, he thinks, prevented by an incission through the muscular coat parallel to the axis of the pylorus. Robin[2] recommends the treatment of ulcer by rectal feeding alone at first, adding a few drops of laudanum to each enema to insure retention, and keeping a wet compress over the stomach. Afterwards he gives nothing but a milk diet for six months, and then gradually changes it to solid food. Hale White[3] remarks that about 45 per cent. of gastric ulcers form adhesions to neighbouring organs, and that it is most important to diagnose the symptoms due to the presence of the latter from those which accompany ulcer or carcinoma. Pain due to adhesions is very long continued, going on sometimes for years, and is not brought on by the ingestion of food. There may be dilatation, as well as adhesions, and in this case we may get vomiting, coated tongue, and other symptoms as well. Adhesions alone may give rise to some local tenderness, and even an indefinite fulness may be felt where the organs are matted together. The main treatment must be by operation and the results are good, but at times the adhesions may re-form, and even if they do not, the patients, having suffered so long, are liable to complain of pains which there is good evidence to show are hysterical or neuralgic. These occasional failures must be considered beforehand in advising an operation, but usually the results are brilliant, and emaciated invalids, crippled by pain, are transformed into persons of vigorous health. The microbic origin of gastric ulcers is advocated by C. B. Box[4] on the grounds of their analogy to other ulcers in the intestinal tube, such as typhoid and dysenteric ones; also from the frequent occurrence of ulcers close together or opposite to each other, the second one being probably an infection from the older one; and finally from the presence of a zone of œdematous inflammatory tissue surrounding an ulcer when in an acute stage. On the other hand ulcers from embolism are difficult to produce experimentally, and very rare in diseases where emboli are most common. If an embolism of the stomach does take place it is usually in a large vessel. As to diagnosis: Hæmatemesis is frequently absent in real cases of ulcer, and on the other hand it is often present with symptoms of dyspepsia in uræmic conditions. Hence the need of careful examinations of the water in all cases of supposed gastric ulcer. Blood is also vomited in the crises of ataxy, in hysterical conditions, and in mitral disease. As an aid to the diagnosis of a perforation in the anterior wall, certain signs at the base of the left lung due to the collection of stomach contents around the spleen are noteworthy. These are diminished movement of the thorax, diaphragm, and abdomen, dulness, with some pain and tenderness, friction sounds, and diminished breath sounds and fremitus. Another spot to which the contents are apt to gravitate is the pelvis, or between the liver and the head of the right kidney.

**Acute Dilatation of the Stomach**, with collapse and copious vomiting, may be, according to Campbell Thomson[5] (*a*) without apparent cause; (*b*) may follow a surgical operation or a heavy meal in weakly persons; or (*c*) may be connected with other stomach lesions. The dilatation and the increased secretion do not always occur together, and the former depends on a paralysis akin to that seen in the intestines during some forms of obstruction. As to treatment, all food should be given by the rectum; the fluid should be drawn off by a stomach tube, and the loss, if great, made up by saline injections, and strychnine should be administered hypodermically. Bradshaw thinks that the cause is pyloric obstruction, otherwise it is hard to see how there could be vomiting, and some of the stomach contents would flow out into the intestines. Thomson, in reply, mentioned dilatation of the heart as occurring without obstruction. He would regard the nerve atony of the walls as primary, and the increased secretion as a secondary dilating force. Four new cases under his own observation were described. Ewart[6] thinks the condition is due to obstruction, not of the pylorus, but at the junction of the jejunum with the transverse part of the duodenum. The primary factor is collapse of the intestine which drags the mesentery like a cord over this part of the duodenum. The treatment may include the use of the knee and elbow position if the patient is strong enough, otherwise he should be turned on the right side, the pelvis raised high and the intestines pressed down from the pelvis towards the thorax. Theodore Fisher and others mention that the duodenum is distended in these cases as well as the stomach, so that it is clear that no pyloric obstruction exists. Box and Wallace[7] think the obstruction is due to the pressure of the stomach on the duodenum where it crosses the spinal column, but that dilatation is primarily due to paralysis. The latter is by no means inconsistent with vomiting, as has been shown by recent physiology. The writers insist on the importance of early diagnosis of this affection, and recommend the treatment proposed by Thomson with the addition of the right-sided or prone posture after syphonage, and the trial of atropine. Ewart suggests the importance of preventing continued dorsal decubitus where there is any danger of the affection.

[1] Brit. Med. Jour., Jan. 18. [2] Med. Rec., Nov. 30. [3] Lancet, Nov. 30. [4] Brit. Med. Jour., Feb. 8. [5] Lancet, Oct. 26. [6] Lancet, Nov. 2. [7] Lancet, Nov. 9.

